# The association of dietary glycemic index and glycemic load with the risk of insomnia in the adult population

**DOI:** 10.1186/s40795-023-00689-x

**Published:** 2023-02-07

**Authors:** Hossein Farhadnejad, Samaneh Sadat, Mitra Kazemi Jahromi, Farshad Teymoori, Asal Neshatbini Tehrani, Ebrahim Mokhtari, Hossein Teymouri, Parvin Mirmiran

**Affiliations:** 1 Student Research Committee, Nutrition and Endocrine Research Center, Research Institute for Endocrine Sciences, Shahid Beheshti University of Medical Sciences, Tehran, Iran; 2 Research Committee and Department of Nutrition, School of Nutrition and Food Science, Isfahan University of Medical Sciences, Isfahan, Iran; 3 Endocrinology and Metabolism Research Center, Hormozgan University of Medical Sciences, Bandar Abbas, Iran; 4 Nutrition and Endocrine Research Center, Research Institute for Endocrine Sciences, Shahid Beheshti University of Medical Sciences, Tehran, Iran; 5 Student Research Committee, Ahvaz Jundishapur University of Medical Sciences, Ahvaz, Iran; 6 Department of Nutrition, School of Allied Medical Sciences, Ahvaz Jundishapur University of Medical Sciences, Ahvaz, Iran; 7 Deputy for Health Affairs, Psychosocial Unit, Tehran University of Medical Sciences, Tehran, Iran

**Keywords:** Glycemic index, Glycemic load, Carbohydrate, Dietary pattern, Insomnia, Adults

## Abstract

**Background:**

A dietary pattern with a high glycemic index (GI) and glycemic load (GL) can be a precursor to sleep disorders that link to many chronic diseases. We aimed to assess the association of dietary GI and GL with the risk of insomnia in Iranian adults.

**Methods:**

A total of 111 newly diagnosed insomnia cases and 333 controls aged 18–60 years were included in this case–control study. The participants’ dietary intakes were collected using a valid and reliable food frequency questionnaire. The diagnosis of insomnia in subjects was performed by a neurologist using the Insomnia Severity Index (ISI) questionnaire. Multivariable logistic regression models, adjusted for the potential confounders, were used to determine the risk of insomnia according to tertiles of dietary glycemic indices.

**Results:**

The mean (SD) age and BMI of the study population (78.6% female) were 31.8 (10.0) years and 24.70 (3.62) kg/m^2^, respectively. The median (IQR) of dietary GI and GL in subjects was 62.7 (57.0–68.6) and 213.5(167.4–268.5), respectively. Based on the multivariable-adjusted model, after controlling for age, sex, physical activity, obesity, smoking, socioeconomic score, general health questionnaire (GHQ) score, and dietary energy intake, the odds of insomnia were increased across tertiles of dietary GL[(OR:2.72,95%CI:1.10–6.70),(P_trend_ = 0.017)], however, no significant association was observed between high GI diet and insomnia risk [(OR:1.38,95%CI:0.77–2.47),(P_trend_ = 0.298)].

**Conclusions:**

Our results revealed that greater adherence to dietary pattern with high GL could be increased the odds of insomnia in Iranian adults.

## Background

Insomnia is the most prevalent type of sleep disorders. It is mainly described as poor sleep quality and quantity and weak daily performance. According to sex, place of living, and explanation of the condition, 10–35% of the adult population is affected by sleep disturbances, and 5–10% meet the insomnia DSM criteria [1–4]. Based on recent studies, the prevalence of sleep disorders in the general population, from consistent problems to periodic patterns, is 9 and 27%, respectively [5]. Insomnia is associated with decreased ability to do tasks, reduced life quality, and increased unfavorable mental and medical outcomes [6]. Also, it has been suggested that insomnia symptoms could be related to an increased risk of mortality [7], cardiovascular diseases [8], and injuries [9].

Recently, studies have mostly focused on the association of nutritional factors with sleep duration and quality [10, 11]. However, results on the role of nutrients in the progression of insomnia symptoms are limited and lower understood. The manipulation of carbohydrate intakes in dietary patterns has received more attention over the years regarding its effect on multiple aspects of sleep. Carbohydrate can be an important nutrient for sleep because it acts as a primary main source of energy for all cells and may be associated with brain function and sleep-related hormonal regulation [12]. An interventional study showed that high carbohydrate (HC) diets significantly decreased slow wave sleep (SWS) and increased rapid eye movement (REM) sleep compared with a low carbohydrate diet or a normal balanced diet [13]. In another study [14], the same changes in SWS observed after consuming an HC test meal. However, some studies revealed that subjects suffering from insomnia had low carbohydrate intakes [12, 15–17].

Moreover, a study conducted on the effect of the glycemic index (GI) on sleep architecture demonstrated significant higher subjective ratings of sleepiness and shortened sleep onset latency after consuming a high-GI meal four h before bedtime compared with a low-GI meal [18]. To the best of our knowledge, evidence on the association between dietary GI and glycemic load (GL) and risk of insomnia symptoms is limited to a large prospective study conducted on postmenopausal women [19]. The Gangwisch et al. study reported that a high-GI dietary pattern may be the main factor for the increased risk of insomnia in women, however, higher adherence to a diet rich in fiber, fruit, and vegetables can reduce the risk of insomnia [19].

Dietary GI and GL are essential indicators for assessing the dietary carbohydrate quality and quantity, which helps us more accurately assess this macronutrient's role in predicting chronic disease risks such as insomnia. Therefore, the present study aimed to identify the possible relationship between dietary GI and GL and the risk of insomnia in Iranian adults.

## Methods and materials

### Study population

The current case–control study was conducted on 444 individuals (111 newly diagnosed insomnia patients and 333 controls aged 18–60 years) referred to Isfahan health centers between July 2016 and August 2017. The cases were recruited among patients with moderate or severe primary insomnia diagnosed by a neurologist using the Insomnia Severity Index (ISI) questionnaire [20, 21]. The participants for the control group were selected among individuals without clinically obvious insomnia. The process of assigning individuals to two control and case groups was as follows, in the health centers of Isfahan city, the referring subjects who were eligible for the study according to the inclusion and exclusion criteria were asked to answer the questions of the ISI questionnaire [20]. According to the ISI questionnaire score, individuals were classified into four levels: severe, moderate, mild, and no insomnia, in terms of the risk of primary insomnia. Next, individuals with moderate and severe risk were categorized as cases, and individuals with mild risk and no insomnia were categorized as controls. Using a simple sampling method, the sampling process continued until the number of cases reached 111 subjects and the number of control reached 333 subjects.

The exclusion criteria of the present study include having heart failure, chronic kidney disease, severe joint diseases, restless leg syndrome (based on self-reported medical history), severe mental disorders [screened by General Health Questionnaire (GHQ) 28 questionnaire], apnea, and sleep-related respiratory disorders (screened by Stop questionnaire), any recent stressful events such as death or a serious illness of a family member, divorce, alcohol consumption, stimulants and sedative drugs intakes, supplement intakes for more than three days a week, shift work, pregnancy, and lactation. We also excluded the individuals who did not respond to more than 35 food items in the food frequency questionnaire (FFQ) or whose caloric intake was outside the range of 800 to 4200 kcal.

### Measurements

#### Assessment of primary insomnia

The presence of primary insomnia in individuals was determined using a brief, reliable, valid self-reporting questionnaire, the ISI [20], which was previously validated among Iranian population [21]. The ISI is a seven-item questionnaire, investigating the nature and symptoms of sleep problems using a Likert-type scale, a type of scale in which respondents specify their level of agreement or disagreement on a symmetric agree-disagree scale for a series of statements. The content of this questionnaire corresponds closely to ICD-10-CM F51.01 and DSM-IV criteria for insomnia. It assesses the individuals’ current understanding of symptom severity [difficulty in initiating sleep (DIS), difficulty in maintaining sleep (DMS), and early morning awakening (EMA)], sleep-related satisfaction, and daytime impairment by seven questions with a 5-point Likert scale (0–4) with a total score of 28. A score of 0–7 represents that individuals have no clinically significant insomnia. The score of 8–14 shows sub-threshold insomnia in participants. The score ranged from 15–21 indicates clinical insomnia with moderate severity, and 22–28 indicating severe clinical insomnia [20]. We classified the case and control groups based on Smith and Trinder's study [22], which suggests a cut-off score of 14 to distinguish individuals with insomnia from normal controls.

#### Dietary assessment

In the current study, participants' dietary intakes were collected using a validated and reproducible 168-item semi-quantitative FFQ [23]. A list of typical Iranian foods with standard serving sizes was included in our FFQ. Individuals were asked to report their average dietary intake during the previous year by choosing one of the following categories: never or less than once a month, 3–4 times per month, once a week, 2–4 times per week, 5–6 times per week, once daily, 2–3 times per day, 4–5 times per day, and six or more times a day. Portion sizes of each food item were converted into grams using standard Iranian household measures [24]. The daily energy and nutrient intakes were computed based on the United States Department of Agriculture’s (USDA) Food Composition Table (FCT). For some local foods unavailable in USDA FCT [25], we used the Iranian FCT [24]. Finally, we used the Nutritionist IV software, modified for Iranian foods, to determine the energy, macronutrients, and micronutrients based on participants’ dietary intakes.

#### Glycemic indices

For each carbohydrate-containing food, GI is described as the area under the blood glucose response curve over two hours after eating the food relative to that after consuming the equivalent amount of carbohydrate as glucose. The international table of GI and list of the GI of Iranian foods [26, 27] were used to obtain the GI value of each food item. The total dietary GI and GL were determined as the following:

$$\mathrm{Dietary}\;\mathrm{GI}=\left[\left(\mathrm{carbohydrate}\;\mathrm{content}\;\mathrm{of}\;\mathrm{each}\;\mathrm{food}\;\mathrm{item}\right)\times\left(\mathrm{number}\;\mathrm{of}\;\mathrm{servings}/\mathrm d\right)\times\left(\mathrm{GI}\right)\right]/\mathrm{total}\;\mathrm{daily}\;\mathrm{carbohydrate}\;\mathrm{intake}$$

$$\mathrm{Dietary}\;\mathrm{GL}=\lbrack\left(\mathrm{carbohydrate}\;\mathrm{content}\;\mathrm{of}\;\mathrm{each}\;\mathrm{food}\;\mathrm{item}\right)\times\left(\mathrm{number}\;o\;\mathrm{fservings}/\mathrm d\right)\times\left(\mathrm{GI}\right)\rbrack/100$$

#### Anthropometrics’ measurements

We measured the participants’ weight to the nearest 100 g with minimum clothes and without shoes using digital scales (model 707, Seca, Hamburg, Germany). A stadiometer (model 208 Portable Body Meter Measuring Device; Seca) was used to measure height to the nearest 0.5 cm in the standing position while the individuals were unshod. Body mass index (BMI) was calculated as weight (kilograms) divided by height (meters^2^).

#### Assessment of other variables

Data on age, sex, marital status, socioeconomic status (SES), medical history, drug use, and smoking status were obtained using a demographic questionnaire. SES score was calculated based on three variables, including household size (≤ 4, > 4 people), education levels (academic and non-academic education), and acquisition (house ownership or not). For each of these variables, participants were given a score of 1 (if their family members were ≤ 4, were academically educated or owned a house) or given a score of 0 (if their family members were > 4, or had non-academic education, or leasehold property). Then, the total SES score was computed by summing up the assigned scores (minimum SES score of 0 to maximum score of 3). An SES score of 3 equated to high, 2 was moderate, and 1 or 0 was low. Through face-to-face interviews, physical activity levels were measured using the international physical activity questionnaire (IPAQ). All results of the IPAQ were expressed as Metabolic Equivalents per week (METs/week) [28].

Considering that severe depression and apnea may lead to secondary insomnia, individuals were screened based on the GHQ28 and Stop questionnaires. GHQ28 had 4 sub-scales for measuring physical complaints, severe depression, anxiety and insomnia, and social dysfunction [29]. Each sub-scale includes 7 questions with 4 replies with a cut-off point of 6 for each sub-scale and a general cut point of 24. Based on this valid and reliable questionnaire, individuals who scored more than 6 for the subscale of depression were excluded from the study. The Stop questionnaire, considered for assessing the obstructive sleep apnea (OSA) risk, consists of 4 questions with two answers: yes or no, which are scored as 0 and 1. Based on the cut-off point for this questionnaire, individuals with a score equal to or greater than 2 were considered at high risk for OSA [30] and were excluded from the study.

### Statistical analysis

Statistical analysis was performed using Statistical Package Software for Social Science, version 21 (SPSS Inc., Chicago, IL, USA). The variables' normality was checked using the Kolmogorov–Smirnov test and histogram chart. Baseline characteristics and dietary intakes were expressed as mean ± SD or median (25–75 interquartile range) for quantitative variables and percentages for qualitative variables. Independent sample t-test and chi-square were used for testing the differences between cases and controls for continuous and categorical variables, respectively (Table 1). Participants were categorized into tertiles based on the dietary GI and GL. The general and dietary data were expressed across tertiles of dietary GL. Linear regression and chi-square tests were used to test the trend of continuous and categorical variables across tertiles of dietary GL (Table 2). We used the logistic regression analysis to determine the odds of insomnia across dietary GI and GL tertiles. The analysis was adjusted for potential confounders, including age and sex, obesity, physical activity, smoking, SES, dietary intake of energy, and GHQ score. The odds ratio (OR) with a 95% confidence interval (CI) of insomnia across tertiles of dietary GI and GL were reported (Table 3), and p-values < 0.05 were considered statistically significant.

## Results

The mean (SD) age and BMI of the participants (78.6% female) was 31.8 (10.0) years and 24.70 (3.62) kg/m^2^, respectively. The median (IQR) of dietary GI and GL were 62.7 (57.0–68.6) and 213.5(167.4–268.5), respectively. Based on Table 1, our findings showed that participants in the cases group had a higher BMI, age, GHQ score, and ISI score than the control group (*P* < 0.05). Also, the protein intake in the case group was significantly lower than in the control group (*P* < 0.05). However, our findings indicated no significant difference in physical activity, % of smoking, % of females, SES score, and the intakes of energy, total fats, and carbohydrates among participants based on case and control groups.

**Table 1:** Study participants' general characteristics and dietary intake based on case and control groups

Variables ^a^	Population		*P*-Value†
	**Case (*n* = 111)**	**Controls (*n* = 333)**	
Glycemic index	64.08 ± 11.85	64.00 ± 19.07	0.968
Glycemic load	230.2 ± 86.7	225.0 ± 85.7	0.580
Age(year)	35.0 ± 10.8	30.7 ± 9.4	**< 0.001**
Female, n (%)	84 (75.7)	265 (79.6)	0.229
Overweight and obese, n (%)	12 (10.8)	16 (4.8)	**0.025**
Smoking, n (%)	4 (3.6)	8 (2.4)	0.350
Physical activity (MET/min/week)	750.0 (240.0 – 1680.0)	792.0 (353.0 – 1700.0)	0.440
SES			0.557
Low	49 (44.2)	153 (46.0)	
Middle	28 (25.2)	90 (27.0)	
High	34 (30.6)	90 (27.0)	
GHQ	20.9 ± 7.4	15.1 ± 6.5	**< 0.001**
ISI score	17.6 ± 2.9	7.4 ± 3.9	**< 0.001**
**Nutrient Intake**			
Energy (Kcal/d)	2387 ± 732	2446 ± 732	0.467
Carbohydrate (% of energy)	60.1 ± 8.5	58.7 ± 8.4	0.115
Protein (% of energy)	13.4 ± 2.6	14.0 ± 2.8	**0.047**
Fat (% of energy)	28.6 ± 7.8	29.3 ± 9.2	0.517

*SES* Socioeconomic status, *IS*I Insomnia severity index, *GHQ* General health questionnaire

^a^Data are presented as mean ± SD or median (25–75 interquartile range) for continuous variables and numbers and percentages for categorical variables

^†^P-Value was computed using an Independent tow sample T-test and Chi-square test for continuous and categorical variables, respectively

Table 2 reported the study participants' general characteristics and dietary intake based on tertiles of the GI score. Participants in the highest tertile of GI were low smoked and had low SES and a lower intake of protein (% of energy) compared to those in the lowest tertile of dietary GI (*P* < 0.05). However, there were no significant differences in other variables, including age, gender distribution, % of obesity and overweight, physical activity, GHQ, ISI score, and intake of energy, fats, and carbohydrate across the tertile of dietary GI.

**Table 2:** The study participants' general characteristics and dietary intake based on tertiles of the glycemic index score^a^

	**Tertiles of GI**			***P*-trend**^†^
Variables ^a^	**T1(*n* = 148)**	**T2(*n* = 148)**	**T3(*n* = 148)**	
Age(year)	32.1 ± 10.1	31.1 ± 9.9	32.0 ± 9.9	0.929
Men, n (%)	25 (16.9)	29 (19.7)	41 (27.7)	0.067
Overweight and obese, n (%)	68 (45.9)	72 (49.0)	64 (43.2)	0.613
Smoking, n (%)	9 (6.1)	-	3 (2.0)	**0.001**
Physical activity (MET/min/week)	817 (396—188)	654 (200—1356)	970 (430—2569)	0.078
SES				**0.016**
Low	36 (24.3)	39 (26.5)	56 (37.8)	
Middle	56 (37.8)	56 (38.1)	54 (36.5)	
High	56 (37.8)	52 (35.4)	38 (25.7)	
GHQ	17.4 ± 7.1	16.2 ± 7.1	16.0 ± 7.3	0.097
ISI score	10.1 ± 5.7	9.8 ± 5.6	10.0 ± 5.8	0.889
**Nutrient Intake**				
Energy (Kcal/d)	2443 ± 749	2422 ± 642	2427 ± 844	0.856
Carbohydrate (% of energy)	58.0 ± 7.1	56.8 ± 7.7	59.0 ± 7.8	0.967
Protein (% of energy)	13.9 ± 2.9	13.8 ± 2.6	13.1 ± 2.6	**0.003**
Fat (% of energy)	28.0 ± 6.4	29.3 ± 7.3	27.7 ± 7.6	0.962

*SES* Socioeconomic status, *ISI* insomnia severity index, *GHQ* General health questionnaire

^†^P for trend was computed using linear regression and Chi-square test for continuous and categorical variables, respectively

^a^Data are presented as mean ± SD or median (25–75 interquartile range) for continuous variables and numbers and percentages for categorical variables

General characteristics and nutrient intakes of the study population across tertiles of dietary GL are shown in Table 3. Participants in the highest tertile of GL were significantly low-active and had lower fat and protein intake compared to those in the lowest tertile of GL (*P* < 0.05). Also, participants in the highest tertile of GL had a higher percentage of males, ISI score, and higher intake of energy and carbohydrate intake compared to those in the lowest tertile of GL (*P* < 0.05). However, there were no significant differences in other variables.

**Table 3:** The study participants' general characteristics and dietary intake based on tertiles of the glycemic load score^a^

Variables ^a^	**Tertiles of GL**			***P*-trend**^†^
	**T1(*n* = 148)**	**T2(*n* = 148)**	**T3(*n* = 148)**	
Age(year)	32.8 ± 10.4	31.8 ± 10.2	30.8 ± 9.4	0.083
Men, n (%)	14 (9.5)	28 (18.9)	53 (36.1)	**< 0.001**
Overweight and obese, n (%)	57 (38.5)	70 (47.3)	77 (52.4)	0.053
Smoking, n (%)	2 (1.4)	6 (4.1)	4 (2.7)	0.342
Physical activity (MET/min/week)	1059 (480—2481)	675 (238.5—1440)	750 (305—1565)	**0.039**
SES				0.115
Low	52 (35.1)	36 (24.3)	43 (29.3)	
Middle	44 (29.7)	65 (43.9)	57 (38.8)	
High	52 (35.1)	47 (31.8)	47 (32)	
GHQ	16.3 ± 6.9	17.1 ± 7.2	16.4 ± 7.5	0.966
ISI score	9.6 ± 5.7	9.3 ± 5.4	11.1 ± 6.0	**0.023**
**Nutrient Intake**				
Energy (Kcal/d)	1827 ± 480	2412 ± 437	3215 ± 625	**< 0.001**
Carbohydrate (% of energy)	56.0 ± 8.9	57.6 ± 6.8	60.4 ± 6.5	**< 0.001**
Protein (% of energy)	14.6 ± 3.2	13.5 ± 2.2	12.9 ± 2.6	**< 0.001**
Fat (% of energy)	29.4 ± 8.1	28.9 ± 6.8	26.7 ± 6.4	**0.005**

*SES* Socioeconomic status, *ISI* Insomnia severity index, *GHQ* General health questionnaire

^†^P for trend was computed using linear regression and Chi-square test for continuous and categorical variables, respectively

^a^Data are presented as mean ± SD or median (25–75 interquartile range) for continuous variables and numbers and percentages for categorical variables

The association between the higher dietary GI and GL and the risk of insomnia is reported in Table 4. In the crud model, there was no significant relationship between GI (OR = 1.10; 95% CI: 0.66–1.84, *P* for trend = 0.676) and GL (OR = 1.32; 95% CI: 0.79–2.20, *P* for trend = 0.195) and odds of insomnia. Also, in the age and sex-adjusted model, no association was found between GI (OR = 1.10; 95% CI: 0.65–1.86, *P* for trend = 0.704) and GL (OR = 1.34; 95% CI: 0.78–2.30, *P* for trend = 0.209) and risk of insomnia. In the multivariable-adjusted model, after adjustment for age, sex, obesity, smoking, physical activity, dietary energy intake, SES, and GHQ score, the odds of insomnia were increased across tertiles of dietary GL [(OR: 2.72, 95% CI: 1.10–6.70), (*P* for trend = 0.017)], however, no significant association was found between high dietary GI and risk of insomnia [(OR: 1.38, 95% CI: 0.77–2.47), (*P* for trend = 0.298)].

**Table 4:** Odds ratios (ORs) and 95% confidence intervals (CIs) for insomnia based on tertiles of glycemic index and glycemic load

	**Tertiles of Glycemic Indices**			***P* for trend**
	**T1**	**T2**	**T3**	
**Glycemic index**				
Median score	154	213	299	
Insomnia /control	37 / 111	29 / 119	45 / 103	
Crude model	1.00 (Ref)	0.71 (0.41 – 1.23)	1.10 (0.66—1.84)	0.676
Model 1^a^	1.00 (Ref)	0.69 (0.39—1.20)	1.10 (0.65—1.86)	0.704
Model 2^b^	1.00 (Ref)	0.66 (0.37—1.17)	1.12 (0.66—1.91)	0.647
Model 3^c^	1.00 (Ref)	0.77 (0.42—1.41)	1.38 (0.77—2.47)	0.298
**Glycemic load**				
Median score	54.2	62.8	71.6	
Insomnia /control	39 / 109	30 / 118	42 / 106	
Crude model	1.00 (Ref)	0.73 (0.42—1.26)	1.32 (0.79—2.20)	0.195
Model 1^a^	1.00 (Ref)	0.72 (0.41—1.26)	1.34 (0.78—2.30)	0.209
Model 2^b^	1.00 (Ref)	0.93 (0.49—1.76)	2.65 (1.14—6.20)	**0.014**
Model 3^c^	1.00 (Ref)	0.81 (0.41—1.59)	2.72 (1.10—6.70)	**0.017**

^a^Adjusted for age and sex

^b^Adjusted for model 1 and physical activity, smoking, overweight and obesity (yes, no), and dietary intake of energy

^c^Adjusted for model 2, socio-economic status, and GHQ score

## Discussion

This case–control study suggested that higher adherence to a high GL diet, characterized by higher intakes of foods with added sugars, refined starchy foods, and sweetened beverages, may be associated with an increased risk of insomnia, whereas no significant association was found between high GI and risk of insomnia.

Evidence on the role of carbohydrate intakes, low carbohydrate diet, or dietary GI and GL on the risk of insomnia lacks sufficient consensus on this topic [15, 17–19]. A cross-sectional study conducted on the Japanese adult population has suggested that a carbohydrate intake of less than 50% of total energy can be associated with some insomnia symptoms such as difficulty maintaining sleep [15], however, Kalam et al. study reported that higher adherence to an alternate day fasting combined with a low carbohydrate dietary pattern did not affect sleep quality, duration, and severity of insomnia [17]. Furthermore, another study indicated that consuming a carbohydrate-based high-GI meal with a simple manipulation of food intake (the type of rice) has led to a remarkable shortening of sleep onset latency in young adult subjects [18]. Our findings are nearly comparable with the results of only a large prospective study investigating the association of dietary GI with the risk of insomnia symptoms among postmenopausal women [19]. The Gangwisch et al. study suggested that individuals with a high-GI dietary pattern may be more prone to the development of insomnia. Also, they declared that greater adherence to the dietary pattern, characterized by higher intakes of fiber, fruit, and vegetables, may decrease the risk of incident insomnia [19]. However, we observed remarkable results only in the relationship between high dietary GL and the risk of insomnia, but in the case of high dietary GI, although the reported ORs showed a possible positive effect of this dietary index in increasing the prevalence of insomnia, these results are not statistically meaningful.

The results of studies indicate that both the quantity and quality of carbohydrate intakes are maybe important and effective in reducing insomnia symptoms such as sleep quality and duration (sleep onset latency, difficulty maintaining sleep, difficulty initiating sleep, etc.). Some studies declared that lower carbohydrate intake might worsen insomnia symptoms [15, 16]. However, in another study, the quantity of carbohydrate intake in the diet is not effective in sleep quality, sleep duration, and severity of insomnia in obese adults [17]. Regarding the importance of carbohydrate quality in predicting insomnia symptoms, our study and the Gangwisch et al. study [19] mostly suggested that adhering to a diet with a high GI or high GL can be an important risk factor exacerbating the symptoms of insomnia.

In the current study, the dietary GL (OR: 2.72) had a remarkable power in predicting the increased risk of insomnia, whereas the association of dietary GI with risk of insomnia is not statistically significant; Some reasons can explain the non-significance of the results related to the dietary GI and the risk of insomnia; first, we must note that the reported OR for insomnia based on the dietary GI is clinically remarkable (OR: 1.38), which shows that a high GI diet can predict an increase in the risk of sleep disorders such as insomnia, but in our study, due to the small sample size and insufficient power of the study, these results are statistically insignificant. Also, GI explains how carbohydrates may affect blood glucose levels, whereas GL takes into consideration every component of the food as a whole, giving a more real-life picture of a food's impact on your blood glucose levels [31–33]. In other words, the GL more accurately measures how certain foods will impact your blood glucose levels and insulin secretion by considering the number of carbohydrates in an average serving [34, 35]. As a result, a food pattern with a high GL is likely to lead to mental disorders and sleep disorders through hyperglycemia, increasing basal insulin, and creating a steep insulin peak [36, 37]. However, the high GI food components of individuals in the form of a dietary pattern can be more effective in increasing blood sugar, insulin levels and insulin-related disorders when the GL level of the individual's diet is also high.

Although the conducted studies according to carbohydrates quality and insomnia and its related variables such as sleep onset latency, sleep duration, or continuation indicated ambiguous findings, it seems that a high GL and GI diet serves as a risk factor for increased risk of insomnia [15–17, 19]; Some reasons and mechanisms may explain this negative effect of a high GI and GL diet on sleep disorders. A higher intake of high GL and GI foods in a meal is the main determinant of elevated postprandial glucose levels and the glycemic response, leading to substantial blood glucose fluctuations and postprandial hyperglycemia [36]. Postprandial hyperglycemia from high dietary GI and GL results in compensatory hyperinsulinemia [36]. Hyperinsulinemia drastically reduces the plasma glucose concentration, which may compromise brain glucose, ∼70 mg/dl [38]. This significant change in plasma glucose level stimulates the secretion of anti-insulin hormones, including autonomic counter-regulatory hormones such as glucagon, epinephrine, corticosteroids, and growth hormone [39]. Increased secretion of these stress hormones leads to symptoms such as heart palpitations, brain stimulation, anxiety, sweating, tremor, paresthesia, irritability, and increased appetite in the body that can affect various aspects of sleep and lead to producing arousal from sleep and reducing sleep efficiency [39–41]. Indeed, hyperglycemia in response to a consuming high GI and GL diet or higher carbohydrates intake can initially lead individuals to drowsy and help them to fall asleep [42]; however, the compensatory hyperinsulinemia and counter-regulatory hormone responses can consequently cause insomnia and arousal [40, 41]. Also, sweetened beverages and added sugars foods are important characteristics of a high GI and GL diet may have an adverse effect on sleep quality because higher intakes of simple sugars can contribute to dysregulation of the intestinal microbiome, a maladaptive microbiota imbalance that can remarkably be effective in various aspects of sleep [43]. Furthermore, higher adherence to dietary pattern with high GI and GL may be led to an increase in the stimulation of inflammatory immune responses [44]; these immune responses can play an essential role in increment the risk of insomnia by inducing the production of anti-inflammatory cytokines that act as sleep inhibitors.

The current study has some strengths. To the best of our knowledge, this study is the first study to investigate the association of dietary GI and GL with the risk of insomnia symptoms in the Middle East and North Africa region peoples by controlling the effects of potential main confounding factors. Also, we focused on subjects with a specific sleep disorder considering both genders. We also used validated questionnaires to collect data on the study population's food intake and physical activity levels. However, some limitations of this study deserve to mention. Our study has a case–control design, which does not allow for determining causal relationships. Also, recall bias is unavoidable due to the use of FFQ to collect nutritional information in this study as a case–control study, however, the use of a validated questionnaire minimizes this error. This study, as a case–control study, could be typically prone to selection bias; however, we selected the study population from the health centers of Isfahan city, which were representative of the adults of Isfahan city, so we minimized this error.

Furthermore, we have determined the sleep problems based on self-reported data as a subjective scale, leading to possible misreports and may affect the findings. It seems that objective measurements such as objective measurements, such as polysomnography or multiple actigraphy for the diagnosis of sleep disorders, could have minimized these errors. Finally, despite adjusting several covariates, the current study cannot control all potential confounding, and the effects of some residual confounding factors and unknown confounders may have occurred.

## Conclusions

In conclusion, the current study reported that higher adherence to a dietary pattern with high GL is related to higher odds of primary insomnia. However, no statistically significant association was observed between a high GI diet and the risk of insomnia. It is suggested that more studies with large sample sizes and prospective designs, such as population-based cohort studies, are performed to examine our hypothesis of the relationship between dietary GI and GL and the odds of insomnia. Also, future population-based observational studies could examine the importance of the effect of the timing of the consumption of carbohydrate foods in modulating sleep at night.

## Data Availability

The datasets analyzed in the current study are available from the corresponding author on reasonable request.

## Abbreviations

CI: Confidence interval
DSM: Diagnostic and Statistical Manual of Mental Disorders
FCT: Food Composition Table
FFQ: Food frequency questionnaire
GHQ: General Health Questionnaire
GI: Glycemic index
GL: Glycemic load
HC: High carbohydrate
IPAQ: International physical activity questionnaire
ISI: Insomnia Severity Index
METs: Metabolic Equivalents
OSA: Obstructive sleep apnea
OR: Odds ratio
REM: Rapid eye movement
SWS: Slow wave sleep
SES: Socioeconomic status
USDA: United States Department of Agriculture
